# Endemicity and genetic diversity of Hepatitis delta virus among Pygmies in Cameroon, Central Africa

**DOI:** 10.1186/s13104-022-05976-7

**Published:** 2022-03-03

**Authors:** Yacouba Foupouapouognigni, Jacques Delors Toumansie Mfonkou, Onana Boyomo, Antoine Gessain, Richard Njouom

**Affiliations:** 1 Virology Service, Centre Pasteur of Cameroon, BP 1274, Yaoundé, Cameroon; 2 Laboratory of Microbiology, University of Yaounde I, BP 812, Yaoundé, Cameroon; 3 Laboratory of Pharmacology and Toxicology, University of Yaounde I, BP 812, Yaoundé, Cameroon; 4 Unit of Epidemiology and Physiopathology of Oncogenic Viruses, URA CNRS 3015, Department of Virology, Institut Pasteur, 25-28 rue du Dr Roux, 75724 Paris Cedex 15, France

**Keywords:** Hepatitis D, Genetic diversity, Pygmies, Cameroon

## Abstract

**Objective:**

A single study conducted about three decades ago on hepatitis D virus (HDV) infection among Baka pygmies in Cameroon reported a very high anti-HDV antibodies prevalence of 46%, but HDV genetic diversity has not been studied in this population. The genetic diversity of strains from endemic ancient populations may help to understand the origin and evolutionary history of viruses. This study aimed to investigate the HDV seroprevalence and the genetic diversity in three remote Cameroonian Pygmies with chronic HBV infection.

**Results:**

An unusually high 69% (36/52) level of HDV infection was found among HBsAg-positive pygmies in Cameroon. HDV RNA was detected and sequenced in 38.8% (14/36). The phylogenetic analysis revealed that 9/14 strains (64.3%) were identified and classified as genotype 1 (HDV-1) and 5/14 (35.6%) as genotype 7 (HDV-7), respectively with a bootstrap value of 100%. The further analysis showed the co-circulation of highly diverse HDV genotypes HDV-1 and HDV-7 in this population. These results highlight the endemicity of HDV infection in Central Africa. The highly diverse HDV-1 and HDV-7 in pygmies suggest an African origin of HDV. However, further studies are needed with larger sample size.

## Introduction

Hepatitis delta virus (HDV) is necessarily associated with hepatitis B virus (HBV) infection and tends to cause more severe disease than HBV infection alone [1]. HDV infection occurs worldwide, but its prevalence largely varies between geographic regions. It is highly endemic in the Middle East, the Mediterranean region, the Amazone bassin, and several African countries [2, 3]. Worldwide, over eight phylogenetically distinct genotypes have been revealed from varied regions. Except the ubiquituous genotype 1 (HDV-1), each virus clade has its distinct geographic distribution: HDV-2 is found in Japan, Taiwan, and Russia; HDV-4 in Taiwan and Japan; HDV-3 in the Amazone bassin; and HDV-5, HDV-6, HDV-7 and HDV-8 in Africa [3, 4]. In a recent large representative sample of the adult population of Cameroon, we found an extreme degree of heterogeneity of HDV distribution across Cameroon, ranging from 1 to 54% according to regions, with the highest prevalence found in the Southern and Eastern regions close to the Equator [5]. A single study conducted about three decades ago on HDV infection among Baka Pygmies in Cameroon reported a very high anti-HDV antibodies prevalence of 46%, but HDV genetic diversity has not been studied in this population [6]. In a previous study conducted in 2011 in Bantou population of Cameroon, we found a high genetic diversity of HDV in Cameroon with the circulation of at least 4 genotypes including genotypes 1, 5, 6 and 7 [6]. In a recent study, the HDV genetic diversity was studied using 211 HDV-positive samples obtained from blood donors in retrospective studies conducted between 2010 and 2016 in Douala and Yaounde in Cameroon as well as patients with illness of unknown etiology, antenatal patients, and participants of door-to- door and voluntary testing campaigns recruited from 14 villages and towns in the South region of Cameroon [7]. HDV genotypes 1, 6, 7, and 8 were identified in the study population, indicating a high level of HDV diversity in Cameroon. Genotype 1 was predominant (65.4%), followed by genotype 7 (28.9%), genotype 6 (5.2%) and one sample of genotype 8 (0.5%). Potential recombinant breakpoints as depicted by sequence branching basal to references were assayed with the aid of SimPlot, however, none was found. The genetic diversity of strains from endemic ancient populations may help to understand the origin and evolutionary history of viruses [8]. In the present study, we investigated the HDV seroprevalence and the genotype diversity in three remote Cameroonian Pygmy populations with chronic HBV infection.

## Main text

### Methods

This study was part of a survey on emerging viruses in Pygmies from Cameroon conducted from 2005 to 2008 [9–11]. The geographic localization and the characteristics of the subjects included in this study are described elsewhere [9]. After obtained consent, whole blood was collected in EDTA tube and sample obtained was centrifuged at 2000*g* during 5 min. Plasma isolated was aliquoted and stored at − 80 °C for subsequent handling. The Murex anti-delta assay (Abbott, Wiesbaden, Germany) was employed as described in the manufacturer’s protocol to determine the presence of HDV antibodies (HDV-Ab). In order to characterize HDV samples, RNA was extracted from 140 µl HDV-Ab-positive plasma using the QIAamp RNA minikit (Qiagen, Courtaboeuf, France) and HDV RNA was detected by reverse transcription (RT)-PCR amplification of a 360-bp fragment coding for the small hepatitis delta antigen (*HDV-sHD*) gene as described previously [12]. Amplicons were sequenced bidirectionally with the BigDye Terminator v3.1 Sequencing Kit (Applied Biosystems) using primers described previously [12]. Sequences editing was done using EDITSEQ (software Seqman™ II Lasergene). HDV reference sequences were retrieved from the GenBank database for multi-alignment using the CLUSTAL W method [13] in the MEGA software version 6 [14]. Originally, we used the neighbour-joining method with 1000-bootstrap replicates to generate an extended phylogenetic tree with all known HDV genotypes. Then, the HDV sequence data sets were downsized by omitting most branches containing no studied sequences. For the analysis of the maximum likelihood of phylogenetic relationship, a reduced data set was used whilst considering the best-fit evolution model. In this case, the General Time Reversible with Gamma distribution GTR + G + I) was used with the assumption that a certain fraction of sites was invariable on an evolutionary level (GTR + G + I). The 1000 bootstrap resampling method was used to assess the reliability of the tree nodes. Kimura two-parameter approach was used to determine the genetic distances between the strains.

For this study, 52 of 62 HBsAg-positive pygmies previously detected were available [9].

### Results

**Fig.1:**
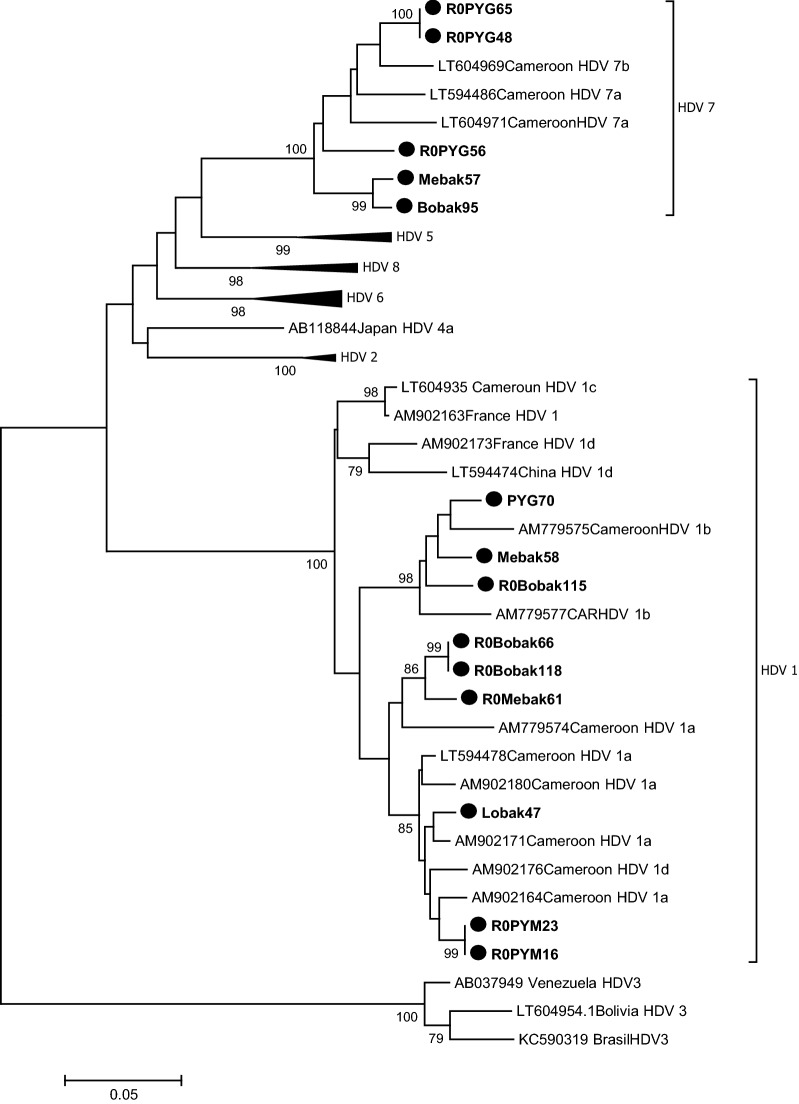
Phylogenetic tree constructed using the *HDV-sHD* nucleotide sequences from the 14 HDV isolates (GenBank accession Numbers: MW773622 to MW773635) from Pygmies (highlighted by a black circle in front of the sample code) and GenBank sequences of HDV-1 to –8 genotypes. Numbers next to the nodes of the tree represent bootstrap values (1000 replicates). Only bootstrap values above 70% are presented

HDV-Ab were found in 36 (69.2%) of the 52 tested plasmas. There was a significant association of HDV-Ab prevalence with age, increasing from 36.6% in children below 10 years of age to 46.2% in 10–20 years old adolescents, and > 75% among the > 21 years old (*P* < 0.001) (Table 1). RNA was detected and sequenced only in 14 out of the 36 HDV-Ab positive samples. The obtained sequences were compared to HDV reference sequences retrieved from Genbank corresponding to the eight HDV genotypes. The phylogenetic analysis revealed that 9 (64.3%) out of the 14 included in this study were classified as genotype 1 (HDV-1) with a bootstrap value of 100% (Fig. 1). The pairwise comparison of these nine strains revealed 81% to 87.1% similarity with a consensus HDV-1 reference sequence. The percentage of similarity was less than 71.9% when compared with HDV-2 to HDV-8 reference sequences (Table 2). Thus, these 9 strains were assigned to HDV-1 genotype. The phylogenetic analyses of the Cameroonian HDV-1 strains identified three clusters (Fig. 1). The first cluster containing three strains (PYG70, Mebak58, and R0Bobak115) was supported by a bootstrap value of 98% and a similarity score of more than 81% was observed between these strains (Table 2). This cluster grouped with two previously described HDV sub-genotype 1b strains from Cameroon and Central African Republic (Fig. 1). The second cluster containing three strains (Lobak47, R0PYM23, and R0PYM16) was supported by a bootstrap value of 85% (Fig. 1) and a similarity score of more than 82% was observed between these strains (Table 2). This second cluster grouped with five previously described HDV sub-genotype 1a strains from Cameroon (Fig. 1). Finally, the third cluster containing three strains (R0Bobak66, ROBobak118, and R0Mebak61) was supported by a bootstrap value of 86% (Fig. 1) and a similarity score of more than 84% was observed between these strains (Table 2). This third cluster did not group with a significant bootstrap with any previously described HDV sub-genotype (Fig. 1). The average nucleotide similarity (83.24%) among the Cameroonian HDV-1 strains is in the same range with the 83% average reported among other worldwide HDV-1 strains [4]. These results also highlight the intra-genotypic diversity of the Cameroonian HDV-1 isolates with the presence of two clearly defined HDV sub-genotypes 1a and 1b and the circulation of three new possible HDV-1 sub-genotypes not yet assigned. The complete HDV nucleotide sequences of these three samples are thus required for a correct assignment. Five strains (35.71%) out of the 14 included in this study (R0PYG65, R0PYG48, R0PYG56, Mebak57, and Bobak95), clustered with previously described HDV genotype 7 (HDV-7) with a 100% bootstrap value (Fig. 1). The pairwise comparison of these five strains revealed a 72.2% to 86.8% similarity with a consensus HDV-7 reference sequence (Table 2). Phylogenetically, these five HDV Cameroonian strains formed three clusters (Fig. 1). The first contained one strain (R0PYG56) with a similarity score of 86.8% when compared to previously described HDV-7 genotype, suggesting that this strain could be assigned to HDV-7 genotype. The second contained two strains (R0PYG65 and R0PYG48), was supported by a bootstrap value of 100% and a similarity score of less than 74% when compared to all 8 previously described HDV genotypes, suggesting that they are distinct from any of the 8 previously described HDV genotypes. The third group contained two strains (Bobak95 and Mebak57) was supported by a bootstrap value of 99% and presented a similarity score of 77.4% and 81% with HDV-7 reference sequence while it was less than 74.6% when compared with the other 7 representatives reference sequences suggesting that they are different from all the 8 previously described HDV genotypes. Although these results do not fulfil the recommendations for a designation of a new genotype (that is, ≥ 3 distinct isolates with high bootstrap values and showing high scores of similarity [4], it is likely that the above strains belong to one or even 2 new HDV genotypes. Further studies in pygmy populations are necessary to get the complete HDV nucleotide sequences of these samples for a correct assignment and to confirm if they could be defined as new HDV genotypes.

**Table 1:** Seroprevalence of HDV-Ab in three pygmy groups of Cameroon infected with HBV

Age	n/N (%; IC95%)			
	Baka	Bakola	Bedzam	Total
≤ 10	1/3 (33.3; − 20–86.6)	3/8 (37.5; 4–71)	0	4/11 (36.6; 8.1–65.1)
11–20	4/7 (57.1; 20.5–93.7)	1/5 (20; − 15.1–55.1)	1/1 (100)	6/13 (46.2; 19.1–73.3)
21–30	8/9 (88.8; 68.2–109.4)	0	2/2 (100)	10/11 (90.9; 73.9–107.9)
31–40	6/6 (100)	0	3/3 (100)	9/9 (100)
41–50	4/4 (100)	0	0	4/4 (100)
> 50	3/4 (75; 32.6–117.6)	0	0	3/4 (75; 32.6–117.6)
Total	26/33 (78.8; 64.9–92.7)	4/13 (30.8; 5.7–55.9)	6/6 (100)	36/52 (69.2; 56.2–81.7)

n: number of HDV-Ab positive samples; N: number of samples tested; %: rate of seroprevalence; IC 95%: 95% confidence interval

**Table 2:** Percent similarity between the R0 gene nucleotide sequences of the 14 HDV samples from Cameroonian Pygmy populations and the representatives of the 8 reference HDV genotypes

	HDV-1	HDV-2	HDV-3	HDV-4	HDV-5	HDV-6	HDV-7	HDV-8
PYG70	82.6	71	60.2	71.1	65.9	66.4	66.4	67.1
R0Bobak-115	83	71.9	60.2	71.2	66.5	66.9	66.6	67.3
Mebak-58	81.6	70.7	60.4	70.3	67.5	67	69.5	68.4
Lobak-47	85	71.3	61.5	70.6	68	66.9	68	68.8
R0PYM16	87.1	72.6	61.8	71.4	69	68.3	68.1	68.6
R0PYM23	82.9	70.7	60.5	68.9	66.5	66.3	66	67.2
R0Bobak-118	85.5	71.4	61.1	71.7	67.4	67.7	67	68.1
R0Bobak-66	85.3	71.8	61.6	71.6	67.2	67.6	67	67.8
R0Mebak-61	84	70.4	60.3	70.1	66.3	66.6	66.2	67.2
R0PYG-56	68	67.9	59.6	69.6	69.9	67.7	86.8	73.6
Bobak95	69	67.9	59.5	69.9	68.5	69	81	74.6
Mebak57	65.5	64.8	57.4	66.4	67.2	66.5	77,4	70.7
R0PYG65	59.6	59.9	54.2	62.6	63	61.4	72.2	65.8
R0PYG48	59.8	60	55.3	62.4	64.4	62.5	73.9	67.4

### Discussion

Our results point to an endemicity of HDV infection in the Cameroonian pygmy populations which is in sharp accordance with data reported from the same population about 30 years ago and to the situation in neighbouring Central African countries. Additionally, HDV infection in Cameroon is characterized by a wide genetic diversity with more than two different genotypes in circulation. This study allows us to highlight possible new HDV genotypes or sub-genotypes obtained from strains isolated from Cameroonian Pygmy populations.

### Conclusion

Our results show an endemic circulation of HDV within the pygmy populations of Cameroon. The description of highly diverse HDV-1 and HDV-7 (African genotype) in this ancient population suggest an African origin of HDV. However, further studies are needed in these groups with larger sample size.

## Limitations

The study of genetic diversity of HDV was carried out on a short sequence (RO). The complete genome would be necessary for assignment of sub-genotype or possibly recombinant forms.

## Data Availability

Nucleotide sequences obtained in the present study were submitted to GenBank under the registration numbers MW773622 to MW773635.

## Abbreviations

HDV: Hepatitis D virus
HBsAg: Hepatitis B surface antigen
ELISA: Enzyme linked immunosorbent assay
